# Prognostic value of “tissue-based” definitions of TIA and minor stroke: Population-based study

**DOI:** 10.1212/WNL.0000000000007531

**Published:** 2019-05-21

**Authors:** Robert Hurford, Linxin Li, Nicola Lovett, Magdalena Kubiak, Wilhelm Kuker, Peter M. Rothwell, for the Oxford Vascular Study

**Affiliations:** From the Centre for the Prevention of Stroke and Dementia, Nuffield Department of Clinical Neurosciences, University of Oxford, UK.

## Abstract

**Objective:**

Since use of diffusion-weighted imaging (DWI) positivity in the “tissue-based” definition of stroke in patients with a clinical TIA is supported by the high associated 90-day risk of recurrent stroke, we aimed to determine long-term prognostic significance, stratified by etiologic subtype, and whether the same tissue-based distinction is predictive in minor strokes.

**Methods:**

Consecutive eligible patients with TIA or minor stroke (NIH Stroke Scale [NIHSS] ≤3) in the population-based Oxford Vascular Study underwent brain MRI at baseline. Stroke risk on 10-year follow-up was stratified by NIHSS (0/1 vs 2/3) and Trial of Org 10172 in Acute Stroke Treatment classification of the initial event.

**Results:**

Among 1,033 patients (633 TIA; 400 minor stroke), 248 (24.0%) had acute lesions on DWI (13.9% of TIAs; 40.0% of minor strokes). A positive DWI was associated with an increased 10-year risk of recurrent ischemic stroke after an index TIA (hazard ratio [HR] 2.66, 95% confidence interval [CI] 1.28–5.54, *p* = 0.009) or a stroke with NIHSS 0–1 (3.03, 1.29–7.08, *p* = 0.011), but not after a stroke with NIHSS 2–3 (0.70, 0.24–2.10, *p* = 0.53). Ischemic stroke risk after DWI-positive TIA was at least equivalent to that after DWI-negative stroke (1.81, 0.82–4.00, *p* = 0.14). Among all patients, DWI positivity was most predictive of 10-year risk after cryptogenic events (4.68, 1.70–12.92, *p* = 0.003).

**Conclusion:**

DWI positivity is associated with an increased long-term risk of recurrent stroke after TIA and minor stroke, supporting a tissue-based definition of minor stroke as well as TIA. Prognostic value is greatest after cryptogenic events.

Use of magnetic resonance diffusion-weighted imaging (DWI) is recommended in investigation of stroke and TIA,^1,2^ and is the basis for the tissue-based definition of TIA as opposed to the traditional time-based definition.^3^ DWI has a higher sensitivity for acute ischemia than plain CT,^4–6^ and an acute DWI lesion is a predictor of 90-day recurrent ischemic stroke following a TIA,^7–11^ independent of the ABCD2 score.^8^

Two other important issues remain to be clarified: whether acute DWI lesions also predict longer-term outcome and whether they predict risk after minor stroke as well as TIA. First, in terms of long-term prognosis, until recently only a few small studies had evaluated the prognostic implications of DWI positivity beyond 1 year, with conflicting results.^12–14^ A recent multicenter registry of nearly 5,000 patients with minor stroke or TIA showed that acute lesions on brain imaging (CT or DWI) were predictive of recurrent ischemic stroke at 90 days but not at 1 year^15^ or 5 years,^16^ although results were not stratified by index stroke vs TIA. Second, although DWI-negative minor stroke is widely recognized,^17^ the prognostic implications remain uncertain.^10,11^ Whether patients and physicians should be reassured by normal imaging even in the presence of focal symptoms and signs lasting longer than 24 hours is unclear. Prognostic uncertainty is often greatest after cryptogenic events, but any difference in prognostic value of DWI between etiologic subtypes of TIA and stroke is also uncertain.

We therefore studied the 10-year risk of recurrent ischemic stroke in DWI-positive vs DWI-negative TIA and minor stroke in a large population-based cohort, stratified according to cryptogenic vs noncryptogenic etiology.

## Methods

We studied consecutive patients referred to the Oxford Vascular Study (OXVASC) with suspected TIA or stroke^18^ between December 2004 and March 2017. OXVASC is an ongoing population-based study of the incidence and outcome of all acute vascular events in a population of 92,728 individuals, registered with 100 general practitioners in 9 general practices in Oxfordshire, United Kingdom. The multiple overlapping methods used to achieve near complete ascertainment of all individuals with TIA and ischemic stroke have been reported previously.^18^ Stroke and TIA were defined according to WHO criteria (acute onset of neurologic deficit, persisting for >24 hours in case of a stroke, or for <24 hours in case of a TIA),^19^ and for the current analyses, we included all patients with DWI performed as part of the initial assessment of TIA or minor ischemic stroke. The closest event prior to the DWI scan was taken as the index event.

Patients were assessed by a neurologist or stroke physician and all presentations and investigations were reviewed by the senior study neurologist (P.M.R.) and TIA or stroke mimics excluded. Demographic data and stroke risk factors were collected from face-to-face interview and cross-referenced with primary care records. Detailed clinical history was recorded in all patients and assessments were made for stroke severity using the NIH Stroke Scale (NIHSS) as recorded on assessment. Minor stroke was defined as NIHSS ≤3. Cause of ischemic events was classified according to the Trial of Org 10172 in Acute Stroke Treatment (TOAST) criteria.^20^

Patients routinely had brain imaging (CT or MRI), vascular imaging (carotid Doppler or CT angiography/MR angiography or digital subtraction angiography), 12-lead ECG, and standard blood tests. Echocardiography, 24-hour ECG (Holter), and 5-day ambulatory ECG monitoring were done when clinically indicated. During the study period, OXVASC brain imaging protocols changed: from 2002 to 2009, CT brain was the first-line imaging modality with MRI for use in selected cases when clinically indicated; from 2009 to 2012, MRI was used routinely for suspected posterior circulation events; and from 2012 onwards, MRI brain became the first-line imaging modality for all patients with suspected TIA or minor stroke. If a patient was reviewed in the emergency department prior to referral to the TIA clinic, he or she would typically receive a CT brain scan acutely followed by an MRI brain scan in the OXVASC clinic. The parameters for MRI scanners and MRI scanning protocols used in the study have been described elsewhere.^21^ Of note, to exclude hemorrhage and mass lesions close to the area of interest on DWI, the study protocol also included a T2-weighted turbo gradient spin echo sequence and T1-weighted imaging. All brain imaging was reviewed by a senior study neuroradiologist (W.K.), blinded to any information about recurrent events. Restricted diffusion (DWI-positive) was defined as hyperintensity on DWI with decreased signal on apparent diffusion coefficient images. Acute DWI lesions were classified as being positive even if they did not correlate with the presenting symptoms.

Patients were followed up face to face at 1, 6, 12, 24, 60, and 120 months by a study nurse or physician to identify any recurrent stroke, supplemented by review of primary care records. Where patients were seen prior to symptom resolution, review at 1 month confirmed a TIA or minor stroke diagnosis. All recurrent events that occurred during follow-up would also be identified by the ongoing daily case ascertainment. Patients who had moved out of the study area were followed up via telephone at the same time points as face-to-face follow-up. We recorded all deaths during follow-up with the underlying causes by direct follow-up, via primary care records, and by centralized registration with Office for National Statistics.

### Statistical analysis

We used Kaplan-Meier survival analysis to calculate the 10-year risks of recurrent ischemic stroke stratified by baseline DWI positivity and compared risks by Cox regression analysis adjusted for age and sex. Predefined subgroup analysis included index event type (ischemic stroke or TIA), NIHSS stroke category (0–1 and 2–3), and TOAST classification^20^ (cryptogenic and noncryptogenic). Analyses were censored at the outcomes of interest: death or the end of follow-up (March 31, 2018). All patients had at least 1 year of follow-up.

Heterogeneity in the prognostic values of DWI positivity was assessed for using the Mantel-Haenszel test. A sensitivity analysis was performed to identify any differences relating to the changes in imaging protocol. All statistical analyses were performed with IBM SPSS version 22.0 (SPSS Inc., Chicago, IL).

### Standard protocol approvals, registrations, and patient consents

Written informed consent or assent from relatives was obtained in all participants. OXVASC was approved by the local research ethics committee (OREC A: 05/Q1604/70).

### Data availability statement

Requests for access to the data reported in this article will be considered by the corresponding author.

## Results

After exclusion of 745 patients referred in the study period with TIA or stroke mimics, 1,033 patients fulfilled the inclusion criteria. Baseline patient characteristics are shown in table 1. There were 400 (38.7%) patients with minor stroke and 633 (61.3%) with TIA. There were 248 patients (24.0%) with DWI-positive lesions: 13.9% of TIAs and 40.0% of minor strokes.

**Table 1:**
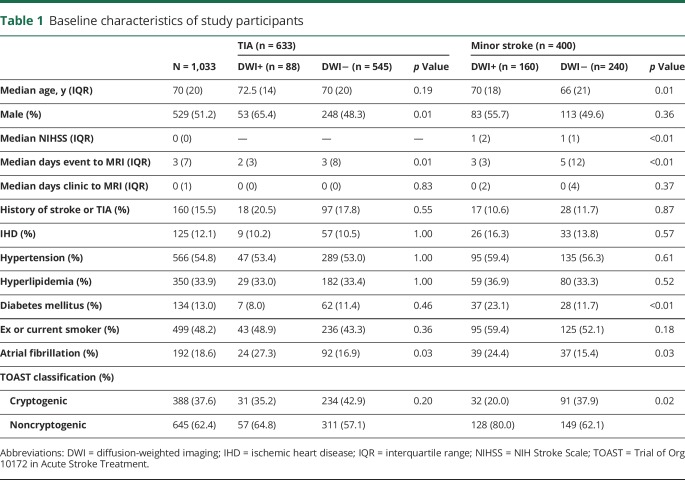
Baseline characteristics of study participants

The median time (interquartile range) from event to scan and clinic assessment to scan was 3 days (7) and 0 days (1), respectively. Among those cases (n = 632/61.2%) investigated after 2012, when the imaging protocol changed to routine use of MRI in all eligible patients, 537 (90.3%) were imaged within 24 hours of assessment.

**Figure 1:**
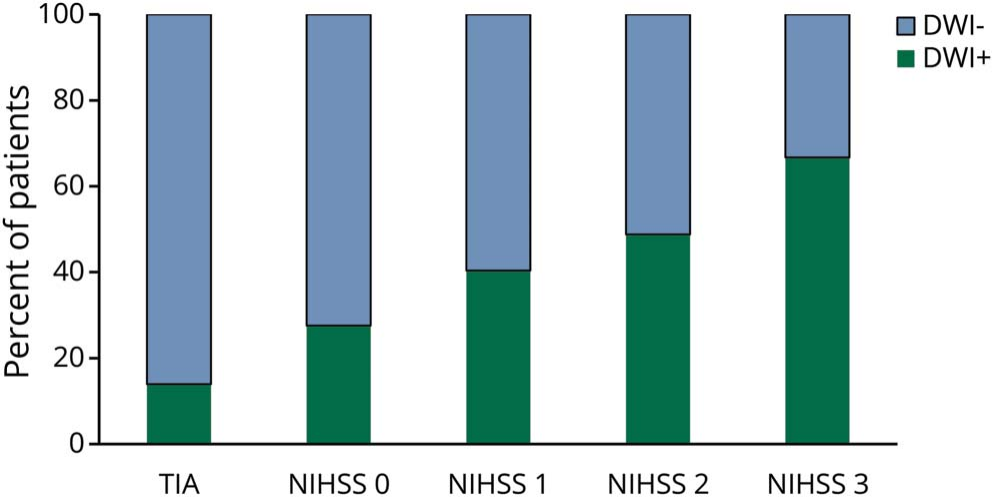
Stacked bar chart depicts the proportion of patients with diffusion-weighted imaging (DWI)+ and DWI− scans categorized by index event type TIA or minor stroke The proportion of patients with acute ischemic lesions on baseline DWI brain imaging (DWI+) and those without (DWI−) categorized by index event type TIA or minor stroke. NIHSS = NIH Stroke Scale.

Figure 1 shows the increasing prevalence of DWI-positive scans with index event severity, ranging from 13.9% in patients with TIA to 65.1% in those with stroke and NIHSS 3.

During 4,778 patient-years of follow-up, there were 70 recurrent ischemic strokes and 141 deaths; 9 and 11, respectively, within 90 days from index event (these cases were excluded from post-90-day analyses). The overall 10-year risks (95% confidence interval [CI]) of recurrent ischemic stroke and death were 9.5% (7.0–12.1) and 22.5% (18.4–26.6), respectively.

**Figure 2:**
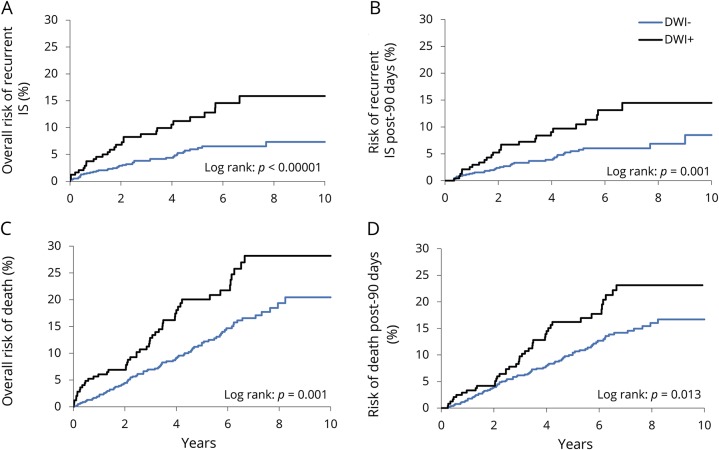
Kaplan-Meier survival graphs for 10-year risks of overall and post-90-day recurrent ischemic stroke (IS) and death The overall 10-year risks and post-90-day 10-year risks of recurrent IS (A, B) and of death (C, D) in patients with acute ischemic lesions on baseline brain diffusion-weighted imaging (DWI+) and in those without (DWI−).

Among all patients with TIA or stroke (figure 2), DWI positivity was predictive of 10-year risks of recurrent ischemic stroke (*p* < 0.00001) and of death (*p* = 0.001). DWI positivity remained predictive for risk of post-90-day recurrent ischemic stroke (14.5% DWI-positive vs 8.9% DWI-negative; hazard ratio [HR] 2.24, 95% CI 1.35–3.72, *p* = 0.002) and death (23.1% vs 16.7%; 1.58, 1.10–2.29, *p* = 0.014). Sensitivity analyses stratified by study phase also showed consistently increased risks in DWI-positive patients with recurrent ischemic stroke (pre-2012 HR 2.00, 1.08–3.71, *p* = 0.03; post-2012 HR 2.70, 1.29–5.65, *p* = 0.008) and death (pre-2012 HR 1.94, 1.24–3.04, *p* = 0.003; post-2012 HR 1.15, 0.86–2.65, *p* = 0.15).

When stratified by type and severity of index event (table 2), there was a trend of increased risk of recurrent ischemic stroke in minor stroke patients (NIHSS 0–3) with a positive DWI (14.9% vs 7.3%; 1.87, 0.96–3.65, *p* = 0.064), driven by those with minor stroke with NIHSS 0–1 (19.2% vs 4.9%; 3.03, 1.29–7.08, *p* = 0.011). These risks remained statistically significant following adjustment for age and sex, and for post-90-day risk. However, a positive DWI was not associated with an increased risk of recurrent ischemic stroke in patients with NIHSS 2–3 (0.70, 0.23–2.01; *p* = 0.53).

**Table 2:**
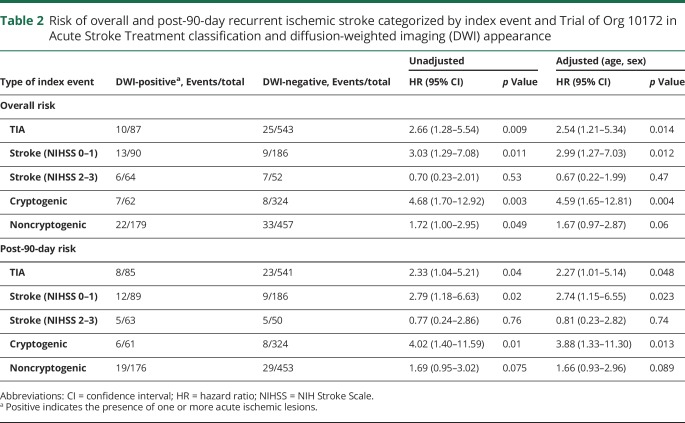
Risk of overall and post-90-day recurrent ischemic stroke categorized by index event and Trial of Org 10172 in Acute Stroke Treatment classification and diffusion-weighted imaging (DWI) appearance

**Figure 3:**
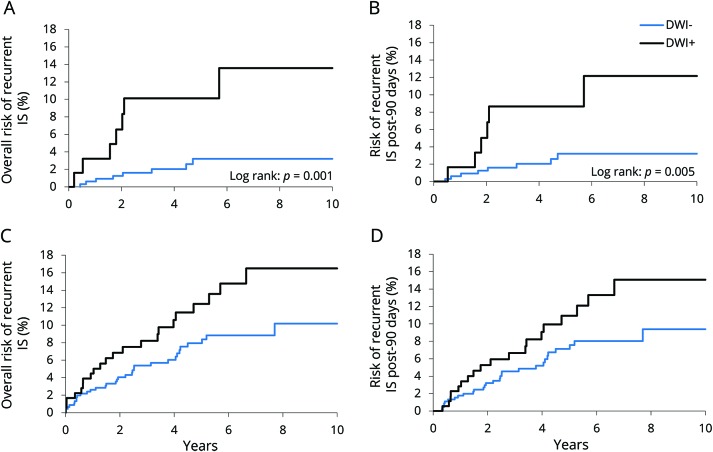
Kaplan-Meier survival graphs for 10-year risks of overall and post-90-day recurrent ischemic stroke (IS) in cryptogenic and noncryptogenic index events The overall 10-year risk and the post-90-day 10-year risk of recurrent IS after cryptogenic (A, B) and noncryptogenic (C, D) index events in patients with acute ischemic lesions on baseline brain diffusion-weighted imaging (DWI+) and in those without (DWI−).

A positive DWI was most predictive of recurrent ischemic stroke in patients with index cryptogenic events, both overall (13.6% vs 3.2%; 4.68, 1.70–12.92, *p* = 0.003) and post 90 days (12.2% vs 3.2%; 4.02, 1.40–11.59, *p* = 0.01; table 2 and figure 3). Findings were similar for the 10-year risk of death in DWI-positive patients in index cryptogenic events (overall 26.7% vs 14.4%; 2.20, 1.05–4.60, *p* = 0.037; post 90 days 20.9% vs 11.2%; 2.08, 0.96–4.49, *p* = 0.06), but not in patients with noncryptogenic event patients (data not shown).

## Discussion

In this large, population-based study with routine MRI, we found that an acute DWI lesion predicted an increased risk of recurrent ischemic stroke after TIA and minor stroke with NIHSS 0–1, and this risk remained elevated for up to 10 years. Furthermore, we identified a particularly elevated risk in those with cryptogenic TIA/stroke etiology.

The rate of DWI positivity (13.9% of TIAs and 65.1% of minor strokes with NIHSS 3) in our study is comparable with previous studies that reported a range between 12% and 67%.^22^ However, the prevalence of positive DWI for TIA in our study was somewhat lower than that reported in previous meta-analyses.^23^ This is perhaps due to the difference in study designs. We chose to include patients with delayed MRI scans up to 1 month postevent, usually due to late presentation, to replicate real-life practice and limit any exclusion bias. Our approach is supported by one study that showed that DWI sequences are still useful in the diagnosis of patients presenting late with TIA or minor stroke (median 17 days), identifying appropriate DWI lesions in 70% of minor strokes and 13% of TIAs.^24^ Due to the delayed scan time, there is a risk of falsely negative DWI; however, despite this potential bias, we have identified a significantly lower risk in these patients, and therefore any reclassification would strengthen the results.

Redefining TIA to a tissue-based (DWI) definition has the benefits of conveying specific prognostic information, but the same approach has not been applied to minor stroke. Our data show that DWI positivity has similar prognostic importance after minor stroke with NIHSS 0–1, which might also help in managing these patients and is reassuring for those with a negative DWI scan. Furthermore, all acute DWI lesions identified in this study were considered, regardless of whether they correlated with the patient's clinical presentation. Our findings would support standard investigation and treatment of such patients with silent lesions, but further studies regarding the long-term risk of asymptomatic DWI lesions would be required.

In addition to the strong existing evidence that TIA patients with an acute ischemic lesion on DWI have a high 90-day risk of stroke, we showed that the prognostic importance of DWI positivity is maintained on long-term follow-up. The reasons why DWI-positive lesions were associated with long-term risks are less clear, although some investigators have postulated that a positive DWI might suggest increased intrinsic vulnerability of the brain to infarction. We found that patients with DWI-positive lesions were more likely to be male, have a noncryptogenic etiology (principally atherosclerotic and cardioembolic; there was a higher rate of atrial fibrillation in DWI-positive patients), and there was a trend to a higher prevalence of diabetes (table 1). The presence of symptomatic intracranial stenosis has also been shown to strongly associate with DWI positivity and recurrent stroke risk in TIA and minor stroke patients.^25^

We showed that DWI positivity is most strongly predictive of recurrent ischemic stroke and death in patients with cryptogenic index events, driven partly by the benign prognosis of patients with DWI-negative cryptogenic events. The lack of a treatable cause of cryptogenic events, such as atrial fibrillation or carotid stenosis, makes secondary prevention in these patients less targeted. The ability to select high-risk cryptogenic TIA/stroke patients on the basis of DWI may allow inclusion into future trials addressing the unmet need for more effective secondary prevention.

Strengths of this study include the large population-based cohort, with stroke-specialist confirmed TIA, blinded radiologic review, and high rates of secondary prevention. However, our study did have some limitations. First, our findings were based on a mainly Caucasian population and may not be generalizable to a more ethnically diverse population. Second, some patients with DWI-negative TIA/minor stroke could have had a nonvascular cause for their symptoms, particularly perhaps those with other normal investigations (i.e., cryptogenic events), but we did use standard diagnostic criteria, administered by an experienced vascular neurologist, and so our results are likely to be generalizable to routine clinical practice. Of note, the 745 patients referred in the study period with a clear nonvascular cause for their symptoms were excluded. Furthermore, we cannot be certain that these results can translate to a hyperacute setting, such as in the emergency department, as our study is a predominantly outpatient, more inclusive cohort.

DWI positivity conveys useful, long-term prognostic information in patients with TIA and minor stroke, supporting the tissue-based definition of TIA, which could also be extended to include those with minor stroke.

## Glossary

CI: confidence interval
DWI: diffusion-weighted imaging
HR: hazard ratio
NIHSS: NIH Stroke Scale
OXVASC: Oxford Vascular Study
TOAST: Trial of Org 10172 in Acute Stroke Treatment

## References

[1] NICE. Stroke and transient ischaemic attack in over 16s: diagnosis and initial management: clinical guideline [CG68]. London: NICE; 2008.

[2] Kernan WN, Ovbiagele B, Black HR, et al. Guidelines for the prevention of stroke in patients with stroke and transient ischemic attack. Stroke 2014;45:2160–2236. 24788967 10.1161/STR.0000000000000024

[3] Albers GW, Caplan LR, Easton JD, et al. Transient ischemic attack: proposal for a new definition. N Engl J Med 2002;347:1713–1716. 12444191 10.1056/NEJMsb020987

[4] Lansberg MG, Albers GW, Beaulieu C, Marks MP. Comparison of diffusion-weighted MRI and CT in acute stroke. Neurology 2000;54:1557–1561. 10762493 10.1212/wnl.54.8.1557

[5] Chalela JA, Kidwell CS, Nentwich LM, et al. Magnetic resonance imaging and computed tomography in emergency assessment of patients with suspected acute stroke: a prospective comparison. Lancet 2007;369:293–298. 17258669 10.1016/S0140-6736(07)60151-2 PMC1859855

[6] Fiebach J, Jansen O, Schellinger P, et al. Comparison of CT with diffusion-weighted MRI in patients with hyperacute stroke. Neuroradiology 2001;43:628–632. 11548168 10.1007/s002340100542

[7] Al-Khaled M, Eggers J. MRI findings and stroke risk in TIA patients with different symptom durations. Neurology 2013;80:1920–1926. 23616156 10.1212/WNL.0b013e318293e15f

[8] Giles MF, Albers GW, Amarenco P, et al. Early stroke risk and ABCD2 score performance in tissue- vs time-defined TIA: a multicenter study. Neurology 2011;77:1222–1228. 21865578 10.1212/WNL.0b013e3182309f91 PMC3179650

[9] Kelly PJ, Albers GW, Chatzikonstantinou A, et al. Validation and comparison of imaging-based scores for prediction of early stroke risk after transient ischaemic attack: a pooled analysis of individual-patient data from cohort studies. Lancet 2016;15:1238–1247. 10.1016/S1474-4422(16)30236-8 27751555

[10] Jing J, Meng X, Zhao X, et al. Dual antiplatelet therapy in transient ischemic attack and minor stroke with different infarction patterns: subgroup analysis of the CHANCE randomized clinical trial. JAMA Neurol 2018;75:711–719. 29582084 10.1001/jamaneurol.2018.0247 PMC5885215

[11] Coutts SB, Simon JE, Eliasziw M, et al. Triaging transient ischemic attack and minor stroke patients using acute magnetic resonance imaging. Ann Neurol 2005;57:848–854. 15929051 10.1002/ana.20497

[12] Purroy F, Montaner J, Rovira A, Delgado P, Quintana M, Alvarez-Sabin J. Higher risk of further vascular events among transient ischemic attack patients with diffusion-weighted imaging acute ischemic lesions. Stroke 2004;35:2313–2319. 15322305 10.1161/01.STR.0000141703.21173.91

[13] Makin SDJ, Doubal FN, Dennis MS, Wardlaw JM. Clinically confirmed stroke with negative diffusion-weighted imaging magnetic resonance imaging: longitudinal study of clinical outcomes, stroke recurrence, and systematic review. Stroke 2015;46:3142–3148. 26419965 10.1161/STROKEAHA.115.010665 PMC4617292

[14] Anticoli S, Pezzella FR, Pozzessere C, et al. Transient ischemic attack fast-track and long-term stroke risk: role of diffusion-weighted magnetic resonance imaging. J Stroke Cerebrovasc Dis 2015;24:2110–2116. 26142258 10.1016/j.jstrokecerebrovasdis.2015.05.016

[15] Amarenco P, Lavallee PC, Labreuche J, et al. One-year risk of stroke after transient ischemic attack or minor stroke. N Engl J Med 2016;374:1533–1542. 27096581 10.1056/NEJMoa1412981

[16] Amarenco P, Lavallée PC, Monteiro Tavares L, et al. Five-year risk of stroke after TIA or minor ischemic stroke. N Engl J Med 2018;379:1580. 10.1056/NEJMc1808913 30332572

[17] Sacco RL, Kasner SE, Broderick JP, et al. An updated definition of stroke for the 21st century: a statement for healthcare professionals from the American Heart Association/American Stroke Association. Stroke 2013;44:2064–2089. 23652265 10.1161/STR.0b013e318296aeca PMC11078537

[18] Rothwell PM, Coull AJ, Giles MF, et al. Change in stroke incidence, mortality, case-fatality, severity, and risk factors in Oxfordshire, UK from 1981 to 2004 (Oxford Vascular Study). Lancet 2004;363:1925–1933. 15194251 10.1016/S0140-6736(04)16405-2

[19] WHO. Cerebrovascular Diseases: Prevention, Treatment and Rehabilitation. Technical Report Series No 469. Geneva: WHO; 1971. 4998212

[20] Adams HPJ, Bendixen BH, Kappelle LJ, et al. Classification of subtype of acute ischemic stroke: definitions for use in a multicenter clinical trial: TOAST: Trial of Org 10172 in acute stroke treatment. Stroke 1993;24:35–41. 7678184 10.1161/01.str.24.1.35

[21] Lau KK, Li L, Lovelock CE, et al. Clinical correlates, ethnic differences, and prognostic implications of perivascular spaces in transient ischemic attack and ischemic stroke. Stroke 2017;48:1470–1477. 28495831 10.1161/STROKEAHA.117.016694 PMC5436733

[22] Adeoye O, Heitsch L, Moomaw CJ, et al. How much would performing diffusion-weighted imaging for all transient ischemic attacks increase MRI utilization? Stroke 2010;41:2218–2222. 20798366 10.1161/STROKEAHA.110.592675 PMC2952926

[23] Redgrave JNE, Coutts SB, Schulz UG, Briley D, Rothwell PM. Systematic review of associations between the presence of acute ischemic lesions on diffusion-weighted imaging and clinical predictors of early stroke risk after transient ischemic attack. Stroke 2007;38:1482–1488. 17379821 10.1161/STROKEAHA.106.477380

[24] Schulz UG, Briley D, Meagher T, Molyneux A, Rothwell PM. Diffusion-weighted MRI in 300 patients presenting late with subacute transient ischemic attack or minor stroke. Stroke 2004;35:2459–2465. 15375305 10.1161/01.STR.0000143455.55877.b9

[25] Coutts SB, Modi J, Patel SK, Demchuk AM, Goyal M, Hill MD. CT/CT angiography and MRI findings predict recurrent stroke after transient ischemic attack and minor stroke: results of the prospective CATCH study. Stroke 2012;43:1013–1017. 22302109 10.1161/STROKEAHA.111.637421

